# Asymmetries of foot strike patterns during running in high-level female and male soccer players

**DOI:** 10.1186/s13102-023-00696-2

**Published:** 2023-07-14

**Authors:** Stanislav Dimitri Siegel, Joel Mason, Daniel Hamacher, Anna Lina Rahlf, Astrid Zech

**Affiliations:** 1 Department of Human Movement Science and Exercise Physiology, Institute of Sport Science, Friedrich Schiller University Jena, Seidelstraße 20, Jena, 07749 Germany; 2 Methods and Statistics in Sports, Institute of Sport Science, Friedrich Schiller University Jena, Seidelstraße 20, Jena, 07749 Germany; 3 Department of Sports Science, Institute of Health, Nutrition and Sport Science, Europa-Universität Flensburg, Campusallee 2, Flensburg, 24943 Germany

**Keywords:** Running, Asymmetry, Foot strike pattern, Sex, Soccer

## Abstract

**Backround:**

Foot strike pattern (FSP) is defined by the way the foot makes initial ground contact and is influenced by intrinsic and extrinsic factors. This study investigated the effect of running speed on asymmetries of FSP.

**Methods:**

Seventeen female and nineteen male soccer players performed an incremental running test on an instrumented treadmill starting at 2.0 m/s until complete exhaustion. Force plate data were used to categorize foot strikes into rearfoot (RFS) and non-rearfoot strikes. Additionally, peak vertical ground reaction force (peakGRF) and stride time were calculated. The symmetry index (SI) was used to quantify lateral asymmetries between legs.

**Results:**

The SI indicated asymmetries of the rate of RFS (%RFS) of approximately 30% at slow running speed which decreased to 4.4% during faster running speed (*p* = 0.001). There were minor asymmetries in peakGRF and stride time at each running stage. Running speed influenced %RFS (*p* < 0.001), peakGRF (*p* < 0.001) and stride time (*p* < 0.001). Significant interaction effects between running speed and sex were shown for %RFS (*p* = 0.033), peakGRF (*p* < 0.001) and stride time (*p* = 0.041).

**Conclusion:**

FSP of soccer players are asymmetric at slower running speed, but symmetry increases with increasing speed. Future studies should consider that FSP are non-stationary and influenced by running speed but also differ between legs.

## Introduction

Foot strike patterns (FSP) are frequently used to describe and assess running gait biomechanics and their adaptability to changing external and internal conditions [1]. Typical characteristics of habitual running patterns can be seen in the moment of foot strike at the beginning of the stance phase [2], with individuals exhibiting rearfoot, midfoot or forefoot strikes. Several authors distinguish only between rearfoot (RFS) and non-rearfoot strike (nRFS) due to similarities of ankle kinetics and muscle activity between midfoot and forefoot strike [3, 4]. The running patterns differ in various biomechanical components [5]. The RFS is generally characterized by a rapid impact transient during the first 50 ms of ground reaction force (GRF) leading to a high vertical loading rate. These impact characteristics can be reduced by an anterior shift of the center of pressure during ground contact [6]. While the RFS leads to a higher load of the patellofemoral joint, the anterior shift increases the load on the ankle and metatarsal joints [7, 8], which may be associated with adaptations of neuromuscular control mechanisms [9].

In the vast majority of studies, between two and eight foot strikes are used to define FSP and to categorize individuals into nRFS or RFS runners, typically captured by high-speed cameras, 3D kinematics or force plates [6, 10, 11]. Although this procedure is widely accepted and replicated, uncertainty remains regarding whether individual FSPs are indeed as stationary as they are currently considered. A leading source of this doubt is evidence that numerous factors can change FSP both transiently and more permanently, which indicates that habitual running patterns may be highly variable in response to external and internal circumstances [12]. A key factor leading to changes in FSP is running speed [13, 14], which is mostly explained by adjustments to higher impact loads and energy costs that occur when the running speed increases. While most studies indicate a nonlinear relationship between changes in FSP and increasing running speed, findings are inconsistent [15, 16]. Considering the variability of FSP during similar external conditions, it seems plausible that this inconsistency arises from the small number of gait cycles that are typically used to determine and categorize individual FSP. However this remains unclear, because only few studies have used more than eight foot strikes for data analysis.

Furthermore, it is also common practice to use single-leg gait cycles to define gait characteristics and FSP [3, 5, 16], which assumes bilateral symmetry of running kinetics and kinematics. Asymmetries in running kinetics [17] and other running related parameters [18] are already mentioned in the literature. However, their relevance and consequences as well as underlying mechanisms are still poorly understood. Since FSP have considerable influence on running kinematics and kinetics it seems reasonable to expect asymmetries in the prevalence of rearfoot and non-rearfoot running patterns as well. Analyzing asymmetries of FSP may also help better to understand the origin of unilateral complaints in running athletes, since FSP can significantly influence loading [19, 20].

The aim of this study was to analyze bilateral FSP at several stages of running speed in highly-trained male and female soccer players. Since men and women seem to differ regarding running biomechanics [21, 22] and risk of injury in running and soccer [23–25] both sexes are equally included and treated differently in the data analysis [26, 27]. A standardized running protocol in a laboratory setting was used in order to obtain a sufficient number of gait cycles needed to account for an individual variability in running patterns at different running speeds. It was hypothesized that 1) asymmetry during all running speeds persists in FSP between the legs, 2) a change of asymmetry would occur with proceed of the running protocol 3) no sex differences exist in asymmetry and FSP.

## Methods

### Study design

A cross-sectional study including a single test session for each participant was performed. Ethical approval was obtained from the local university ethics committee (protocol number FSV 21/003). All participants and, in the case of minors, their parents/legal guardians, gave their written consent to participate in this study and were informed that they could withdraw their participation at any time without giving reasons. During the study process the authors followed the rules of the Helsinki Declaration. The study reports according to the Strengthening the Reporting of Observational Studies in Epidemiology (STROBE) guidelines for reporting observational studies [28].

### Participants

Twenty-one female soccer players from one German Bundesliga team (first division) and nineteen male youth soccer players (first division of the German U19 league) participated in the study. Tests were conducted during the pre-season phase prior to any training session. Exclusion criteria for participation were contraindications related to the incremental running test protocol and surgery or injury in the last two months.

### Instrumentation and test procedure

Participants were instructed to arrive at the laboratory at least three hours postprandial, fully hydrated and to avoid strenuous exercise in the 48 h prior to a testing session. All participants wore their preferred running shoes. All measurements were performed on an instrumented treadmill (Bertec Corporation, USA). Kinetic data were recorded at a sample rate of 1080 Hz. Participants were provided with a safety belt during running. Two video cameras (type Oqus 210c) of the motion capture system (Qualisys AB, Sweden) were used to capture sagittal and frontal lower extremity running movements at 120 Hz. To quantify the lactate concentration, ear blood samples was analyzed with a lactate and glucose analyser (Biosen C-Line, EFK Diagnostics, Germany) at each running stage.

At the beginning of the measurement, weight, height and leg dominance were collected. Leg dominance was defined by asking participants which leg they use to kick a ball [29]. Resting ear blood samples were then obtained after sitting quietly on a chair for 5 min. The treadmill running protocol was designed following the previously published guidelines [30]. Warm-up and familiarization consisted of a 3-min run on a low intensity, comfortable (self-selected) speed. The speed of the treadmill was increased by 0.5 m/s at each stage which lasted 3-min until the tester had to stop the running test due to a termination criterion (e.g. subject cannot continue running). At the end of each running stage, the treadmill was stopped to allow blood sampling and simultaneous recording of the subjective exertion using the rate of perceived exertion (RPE) scale. Each resting period between stages was no longer than 60 s.

### Data analysis

Data of the force plates were captured and exported to a text file with the Qualisys Track Manager (Version 2019.2, QTM, Gothenburg, Sweden). Data processing was done in Matlab (Mathworks, USA). The data were filtered using a 7th order, zero-lag Butterworth low pass filter with a cutoff frequency of 65 Hz. After removing the first 10 steps at each running stage, the first 2 min of each stage were used for data analysis.

**Fig. 1:**
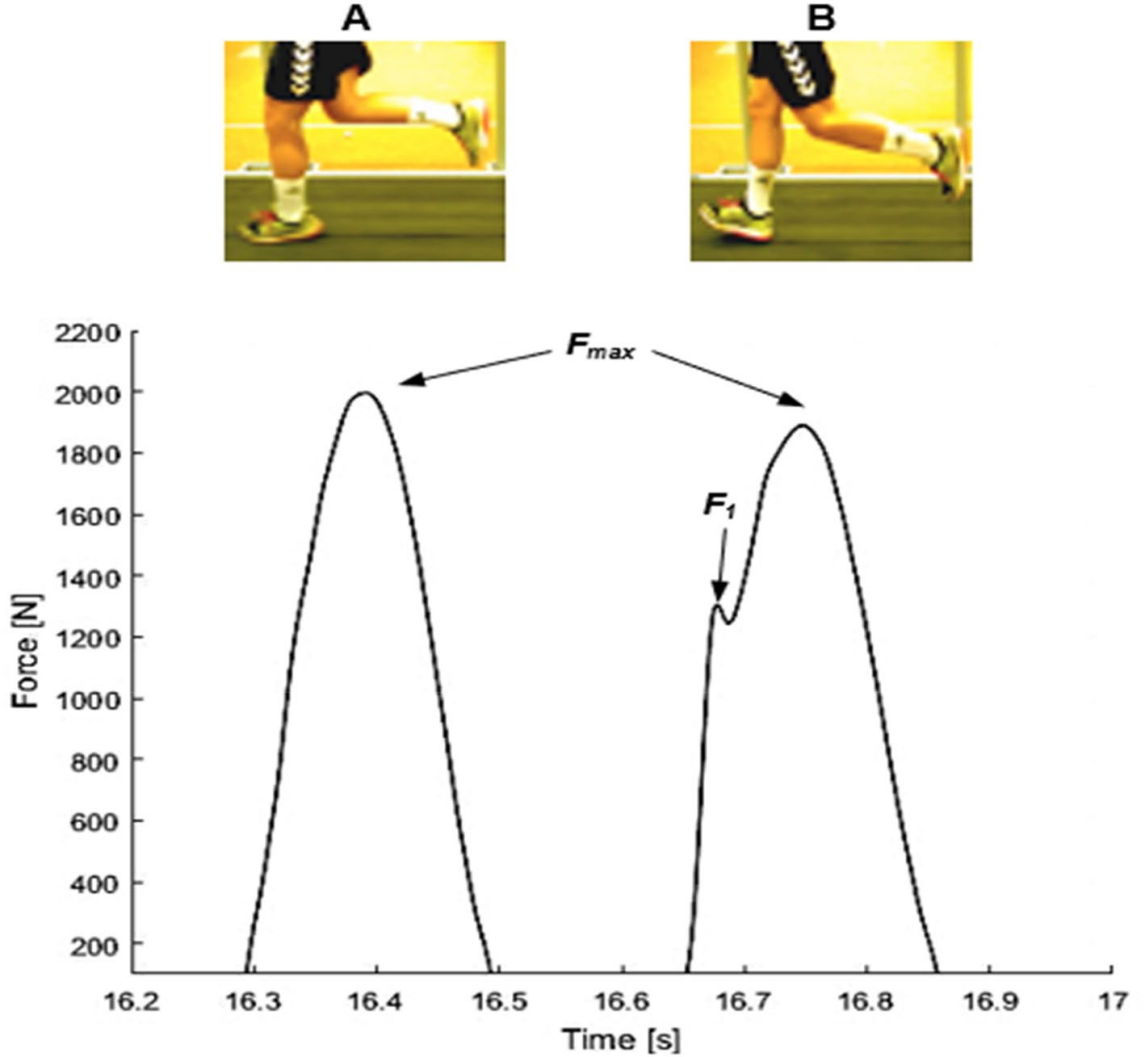
Example of a force-time curves and image captures of two consecutive foot strikes from an athlete/subject in stage 3, (**A**) nRFS of the left leg and (**B**) RFS of the right leg. The foot strikes were classified by the presence of an impact transient (F1). Fmax denotes the maximum ground reaction force

Foot contacts were identified by a GRF greater than 100N [31]. Stride time was defined as the time between two consecutive heel strikes by the same leg. The maximum vertical GRF (peakGRF) of each step was determined as the highest value of the force–time curve. For each running stage, the mean value and the standard deviation of the stride time as well as the peakGRF were calculated. For comparison between subjects, peakGRF values were normalized to body weight of each subject. FSP were determined by using force curves of each stance phase and validated through visual analysis of laterally and posteriorly placed video cameras. A RFS was identified by the presence of an additional peak in the GRF curve (impact transient). The absence of an additional peak indicated a nRFS (Fig. 1) [6, 32]. For each individual, the rate of RFS (%RFS) at each running stage was calculated by using all gait cycles of the two-minute running periods. Therefore, the closer the value was to 100%, the more RFS were used.

To analyse asymmetries between the left and right leg for each outcome the symmetry index (SI) was calculated using the following modified formula according to the method of Robinson*, *et al*.* [33]:

$$SI= \frac{|({x}_{right} - {x}_{left})|}{\left(\frac{1}{2}\right)({x}_{right} + {x}_{left})} \times 100\%$$

For ${x}_{right}$ the relative number of the RFS, peakGRF and stride time of the right leg and for ${x}_{left}$ the same variables of the left leg of each running stage were inserted into the formula. The SI is a quantitative indicator that gives the percentage difference between a variable measured on the right and the left. The value of SI = 0 indicates full symmetry, while SI ≥ 100% indicates asymmetry [17].

### Statistics

Data were presented as mean and standard deviation (SD) for the absolute variables of %RFS, peakGRF, and stride time as well as the SI of %RFS, peakGRF, and stride time. Sex differences in participant characteristics (age, height, weight and blood lactate concentration) were tested with the one-factor ANOVA. The effects of the factors running speed and sex, and their interaction on the outcomes %RFS, peakGRF and stride time were modeled using multi-level model (measurements nested within leg side nested within participants) with random intercepts. The effects of running speed and sex, and their interaction on SI were analyzed using a two-factor repeated-measures ANOVA. Greenhouse–Geisser corrections were applied when sphericity was not met according to Maulchy’s Test of Sphericity. An a priori alpha level of *p* < 0.05 was used to qualify statistical significance for all analyses.

## Results

A total of 17 females (age 21.5 ± 4.3 years; height 1.7 ± 0.1 m; weight 65.2 ± 7.5 kg; BMI 22.9 ± 1.6 kg*m^−2^) and 19 males (age 16.9 ± 0.8 years; height 1.76 ± 0.06 m; weight 73.4 ± 7.0 kg; BMI 23.4 ± 1.2 kg*m^−2^) were included in data analysis. Four female individuals were excluded because they did not complete the 6th running stage. The 6th stage was defined as the cut-off because this stage included the highest running speed, which was completed by 90% of the subjects, while only 60% of the subjects were able to complete the 7th stage. The right leg was the dominant leg in 29 participants and the left leg in 7 participants. Significant differences between female and male soccer players were found for age (*p* = 0.001), height (*p* < 0.001) and weight (*p* = 0.001) as well as for blood lactate concentrations at higher running speed (Table 1).

**Table 1:** Sample mean characteristics (± SD) and ANOVA statistics for differences between females and males

	**Females (*n* = 17)**	**Males (*n* = 19)**	***P*-value**
Age (y)	20.52 (4.40)	16.89 (0.81)	0.001
Height (m)	1.69 (0.05)	1.77 (0.06)	< 0.001
Body weight (kg)	65.21 (7.46)	73.38 (7.07)	0.001
BMI (kg*m^−2^)	22.87 (1.66)	23.42 (1.23)	0.240
Blood lactate (mmol/l)			
Stage 1 (2.0 m/s)	1.41 (0.37)	1.34 (0.23)	0.234
Stage 2 (2.5 m/s)	1.58 (0.47)	1.41 (0.31)	0.060
Stage 3 (3.0 m/s)	2.18 (0.68)	1.84 (0.47)	0.021
Stage 4 (3.5 m/s)	3.66 (1.13)	2.68 (0.73)	0.001
Stage 5 (4.0 m/s)	6.65 (1.73)	4.35 (1.30)	< 0.001
Stage 6 (4.5 m/s)	10.51 (1.96)	7.07 (2.02)	< 0.001

*BMI* Body mass index

**Fig. 2:**
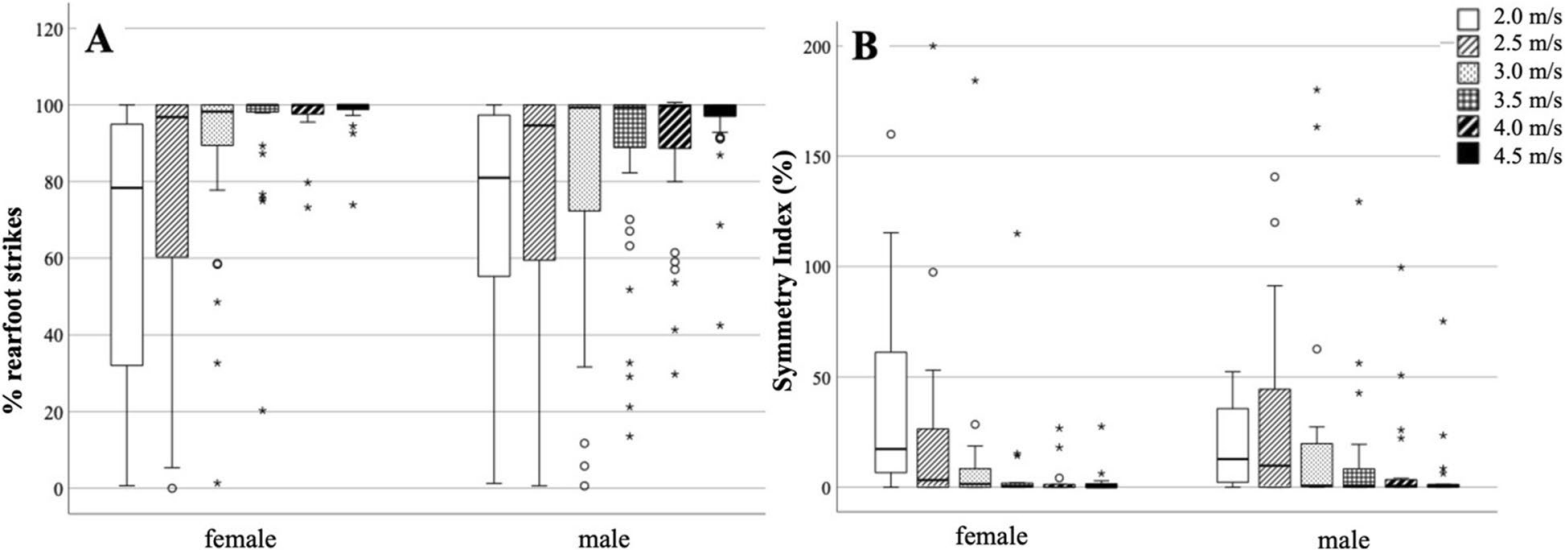
Boxplots for the percentage of rearfoot strikes (%RFS) (**A**) and Symmetry Index (SI) (**B**) at each running speed stage (mild outliers = °, extreme outliers = *)

Changes in %RFS, as well as SI of %RFS throughout the running stages for female and male participants, are shown in Fig. 2. The multi-level model with random intercepts Table 2 revealed a significant effect of running speed on the %RFS (*p* < 0.001), peakGRF (*p* < 0.001), and stride time (*p* < 0.001). While the %RFS as well as the peakGRF increased, the stride time decreased continuously. A significant interaction between running speed and sex was found for the %RFS (*p* = 0.033), peakGRF (*p* < 0.001) and for stride time (*p* = 0.041).

**Table 2:** Mean (SD) for the percentage of rearfoot strikes (%RFS), normalized maximum ground reaction force (peakGRF) and stride time at each treadmill running speed stage, and p-values of the random intercept model

	**Running speed**						**Statistics** **(Analysis of Deviance, type III tests)**		
	**2.0 m/s**	**2.5 m/s**	**3.0 m/s**	**3.5 m/s**	**4.0 m/s**	**4.5 m/s**	**Speed**	Speed*Sex	**Sex**
							P	P	P
**%RFS**									
female	65.1 (34.0)	79.8 (28.2)	87.8 (22.6)	94.4 (15.0)	97.8 (5.7)	98.4 (4.6)	*p* < 0.001	*p* = 0.033	*p* = 0.355
male	71.6 (29.2)	78.3 (31.5)	82.0 (30.6)	86.8 (24.8)	89.9 (18.6)	95.8 (10.6)			
all	68.5 (31.5)	79.0 (29.8)	84.7 (27.1)	90.4 (21.0)	93.6 (14.6)	97.0 (8.4)			
**GRF (N/Kg)**									
female	2.01 (0.32)	2.21 (0.31)	2.21 (0.31)	2.36 (0.30)	2.46 (0.30)	2.53 (0.30)	*p* < 0.001	*p* < 0.001	*p* = 0.467
male	2.11 (0.09)	2.31 (0.11)	2.31 (0.11)	2.48 (0.15)	2.63 (0.17)	2.74 (0.18)			
all	2.06 (0.24)	2.26 (0.24)	2.26 (0.24)	2.42 (0.24)	2.54 (0.26)	2.63 (0.27)			
**Stride time (s)**									
female	0.74 (0.04)	0.72 (0.05)	0.72 (0.04)	0.70 (0.03)	0.67 (0.04)	0.65 (0.04)	*p* < 0.001	*p* = 0.041	*p* = 0.138
male	0.76 (0.06)	0.74 (0.03)	0.73 (0.03)	0.71 (0.03)	0.70 (0.03)	0.68 (0.03)			
all	0.75 (0.05)	0.73 (0.04)	0.72 (0.03)	0.71 (0.03)	0.69 (0.04)	0.67 (0.04)			

Out of the 40 soccer players, *n* = 36 showed asymmetrical FSP at 3.0 m/s. The repeated measures ANOVA determined significant differences in the SI between running stages for the %RFS (*p* = 0.001). The SI decreased as the running speed increased. There was no significant difference in the SI for the peakGRF and stride time between the running stages and the sex (Table 3).

**Table 3:** Mean (SD) for the symmetry index (SI) of the percentage of rearfoot strikes (%RFS), normalized maximum ground reaction force (peakGRF)) and stride time at each treadmill running speed stage, and repeated measures ANOVA statistics (*P*-values and Cohens d)

	**Running speed**						**Statistics**								
	**2.0 m/s**	**2.5 m/s**	**3.0 m/s**	**3.5 m/s**	**4.0 m/s**	**4.5 m/s**	**Speed**			Speed*Sex			**Sex**		
							P	${\eta }^{2}$	F	P	${\eta }^{2}$	F	P	${\eta }^{2}$	F
**%RFS**															
female	37.3 (47.5)	28.1 (51.5)	15.5 (44.2)	9.1 (27.7)	3.1 (7.5)	2.4 (6.6)	0.001	0.174	7.146	0.272	0.038	1.327	0.789	0.001	0.073
male	21.2 (19.9)	31.3 (45.8)	25.8 (53.7)	14.6 (31.8)	11.1 (25.1)	6.3 (17.6)									
all	28.8 (36.1)	29.8 (47.9)	20.9 (49.1)	12.0 (29.7)	7.3 (19.1)	4.4 (13.5)									
**GRF**															
female	0.14 (0.22)	0.14 (0.19)	0.09 (0.18)	0.07 (0.13)	0.10 (0.12)	0.11 (0.21)	0.301	0.035	1.233	0.695	0.012	0.423	0.207	0.046	1.657
male	0.07 (0.06)	0.07 (0.07)	0.06 (0.05)	0.05 (0.04)	0.08 (0.06)	0.07 (0.07)									
all	0.11 (0.16)	0.10 (0.14)	0.08 (0.13)	0.06 (0.10)	0.90 (0.09)	0.09 (0.15)									
**Stride time**															
female	0.010 (0.010)	0.006 (0.006)	0.006 (0.004)	0.006 (0.007)	0.009 (0.007)	0.011 (0.012)	0.132	0.051	1.827	0.169	0.046	1.654	0.597	0.008	0.282
male	0.008 (0.007)	0.007 (0.006)	0.011 (0.009)	0.012 (0.011)	0.009 (0.006)	0.010 (0.008)									
all	0.0085 (0.008)	0.007 (0.006)	0.009 (0.007)	0.009 (0.009)	0.009 (0.006)	0.011 (0.010)									

## Discussion

The main finding of this study is that FSP of high-level soccer players do not only change with increasing running speed, but also differ significantly between legs. While other biomechanical parameters such as the peakGRF and stride time showed only marginal asymmetries, the lateral asymmetry of FSP was 29–30% at the first running stages. However, along with the reduction of the prevalence of nRFS, the asymmetry of FSP decreased with increasing running speed stages. This indicates a high variability and speed-dependency of individual FSP which should be considered in future studies.

### Asymmetry of foot strike characteristics

Only few studies [10, 11, 34] have previously analyzed FSP asymmetries during running in athletes. Two marathon studies [10, 34] identified asymmetric FSP in approximately 4–7% of runners. Due to the small number of foot strikes of each runner included in the analysis and the large number of possible confounding variables resulting from the study design, the results of both investigations should be viewed with caution. Breine, Malcolm, Frederick and De Clercq [11] investigated the FSP of 55 runners at four different speeds and reported, contrary to our findings, an increase in asymmetry with increasing running speed. However, there were considerable differences in the assessments between this and our study. While we used all foot strikes of each two-minute treadmill running stage the participants in the study of Breine, Malcolm, Frederick and De Clercq [11] performed running bouts over a 25-m instrumented walkway at four speeds and GRF curves of only six foot strikes per running stage were used for data analysis. Furthermore, the subjects of all these studies were runners, who are likely to display more consistent and less variable running patterns compared to our cohort of soccer players due to experience, exposure and coaching factors. It can be assumed that soccer players prefer the dominant leg for manipulative motor tasks and the non-dominant leg for stabilizing tasks [17]. The cause of lateral task discrimination is seen as the complex interaction of neurophysiological mechanisms and cortical components including both brain hemispheres [35]. Lateral preferences of foot strike characteristics in soccer players are also probably associated with other asymmetries such as an imbalance of leg strength [36], mobility [37], and in biomechanical and performance related parameters of other movement tasks [38].

The observed changes of FSP asymmetries with increasing stages of the running protocol may be due to two factors: running speed and fatigue. Blood lactate concentration was used to estimate the state of exhaustion [39]. Based on the changes in lactate data with increasing running intensity we assume that all participants were considerably fatigued at the 6th stage of the incremental test. Although other studies have hypothesized that fatigue increases asymmetries between limbs [40], the FSP in our study became more symmetrical with increasing fatigue. Asymmetry and high limb variability are associated with a reduction in performance and additional metabolic costs [41, 42]. Radzak*, *et al*.* [43] investigated the influence of fatigue on lower limb asymmetries in 20 healthy individuals during running and observed higher asymmetry in internal rotation and stiffness of the knees in the fatigued state accompanied by a slight reduction of asymmetry in vertical stiffness, loading rate and joint moments. Another study from Gao*, *et al*.* [44] came to a similar conclusion, indicating that fatigue initiates a change of symmetry which depends on the examined variable.

In our study, the change in FSP and its asymmetries may also be a result of an increasing running speed. Studies on the influence of running speed on lateral symmetry are inconsistent. While Cavagna [45] reported in agreement with our findings an increased symmetrical landing and take-off pattern during running at higher velocities, other studies [46, 47] observed no changes during running. Possible explanations for a more symmetrical FSP at faster speed could be an increased isometric muscle work during landing and take-off, allowing the tendon to almost completely absorb the workload [45].

### Changes in foot strike patterns

The increasing symmetry was associated with a general increase in the percentage of RFS. Hanley, Bissas, Merlino and Gruber [34] and Larson, Higgins, Kaminski, Decker, Preble, Lyons, McIntyre and Normile [10] investigated the FSP of elite and recreational runners at specific distance locations during a marathon using video analysis. In agreement with our findings, both groups of authors reported a clear majority of RFS at all distances. The prevalence of RFS increased in both studies by 6–16% between the first and the last locations. Since the running pace during a marathon remains almost steady throughout the race in most runners the increase of RFS in both studies is conceivably driven by fatigue. Considering that we also observed an increase in the prevalence of RFS, we assume that besides running speed, the increasing fatigue is at least partly responsible for the change in FSP.

A possible explanation for the adjustment of FSP is to reduce the energy cost as well as the load at the moment of ground contact [6, 48]. The anterior shift in the point of force application during foot strike may be due to an anterior tilt of the foot with increasing running speed [13, 14]. However, this relationship was only partially confirmed in other studies [11, 15, 16]. The results of the present study showed an increase of %RFS by 28.5% along with the increasing running speed. In addition, an interaction effect between running speed and sex in %RFS was observed, with females showing a smaller %RFS only in the first stage. Preservation of the FSP may be an involuntary strategy of the human body to maintain stability during running [49]. Another explanation is neuromuscular fatigue. During long distance running, a change in the FSP to a more posterior foot strike was observed after 19 km compared to 10 km [34]. The overall higher %RFS in women, except the first running stage, could be explained by higher fatigue at the same running speed stage, with one indicator being higher lactate concentrations.

### Changes in peakGRF and stride time

Furthermore, an interaction effect between running speed and sex in stride time and peakGRF was observed. There is little evidence for sex differences in running. A possible explanation for these results could be different speed maintenance strategies. For example, due to the smaller height of the female subjects, they may maintain their running speed with a higher stride frequency, resulting in a lower stride time, while the male subjects may have a longer stride length [50]. A higher stride frequency and thus a shorter stride length at the same running speed is associated with a lower peakGRF and vice versa [51].

## Limitations

The main limitation of the study is the application of a treadmill. Running on the treadmill could have both promoted and reduced gait asymmetry, depending on the subjects’ level of experience [52]. The use of a treadmill makes it possible to keep the running speed constant, which lead to less movement variability and thus to a more symmetrical running pattern [53]. The treadmill could also have manipulated the FSP due to the cushioning system [54]. The results of the SI must also be interpreted with caution, as the SI may indicate overestimated values in the case of clinically irrelevant differences between the sides. Further, due to the longer running times at each running stage there might have been an overlap of effects of running speed and fatigue. However, major changes in FSP symmetry were observed the first running stages with lower blood lactate concentrations suggesting that speed plays a considerable role in the change in FSP.

## Conclusion

The present study investigated the adaptation of the FSP of both legs of high-level soccer players during an incremental running protocol. An increase in the %RFS with increasing running speed and fatigue was observed. In addition, asymmetries were found in the FSP, which became more symmetrical with increasing running speed despite fatigue. Since the majority of running in a soccer game occurs at a jogging or walking pace [55], the results of this study have particular relevance to the population studied. Future studies should take these results into account and include both legs in their analysis due to possible asymmetries.

## Data Availability

The raw data sets generated and/or analyzed during study are not publicly available due to the agreement with the soccer club. For further information regarding the data sets, please contact the corresponding author.
